# Safety Precautions for Self-Performed Severe Acute Respiratory Syndrome Coronavirus 2 Tests: A Case of a Swallowed Swab

**DOI:** 10.7759/cureus.15297

**Published:** 2021-05-28

**Authors:** Dávid Molnár, Ferenc Zsigmond, Frigyes Helfferich

**Affiliations:** 1 Department of Otorhinolaryngology and Head and Neck Surgery, Medical Centre, Hungarian Defence Forces, Budapest, HUN; 2 Department of Anatomy, Histology and Embryology, Semmelweis University, Budapest, HUN; 3 Department of Gastroenterology, Medical Centre, Hungarian Defence Forces, Budapest, HUN

**Keywords:** sars-cov-2, covid-19, self-testing, computed tomography, gastroscopy, adverse event, foreign body

## Abstract

Oropharyngeal and nasopharyngeal specimens collected by swabbing are the pillars of severe acute respiratory syndrome coronavirus 2 (SARS-CoV-2) diagnostics. Commercially available rapid antigen tests and self-sampling polymerase chain reaction services have made specimen collection available anytime and anywhere in nonmedical settings. In this study, we report the case of a 45-year-old man who accidentally ingested a swab during self-performed SARS-CoV-2 rapid antigen testing. Imaging studies revealed an elongated foreign body in the stomach. Urgent gastroscopy confirmed the presence of the swabbing applicator in the gastric lumen, which was retrieved using a loop without any complications. Millions of SARS-CoV-2 tests are performed daily, of which an increasing proportion are performed by laypeople. Foreign bodies account for a particular set of complications, which can be avoided by cautious sampling and using the correct technique. Radiopaque labeling of instruments would be useful. Otherwise, rare serious events can occur that may require immediate medical interventions.

## Introduction

The coronavirus disease 2019 (COVID-19) has overwhelmed not just medical practice but everyday life, and testing has become a commonplace procedure. The most common types of severe acute respiratory syndrome coronavirus 2 (SARS-CoV-2) diagnostics are viral genome amplification by real-time reverse transcription polymerase chain reaction and immunochromatographic detection of viral antigens. The registered number of performed tests is monitored, but the rate of direct-to-consumer screening options is also increasing [1].

Nasopharyngeal (NPS) and oropharyngeal swabs (OPS) are the mainstays of sampling for either laboratory tests or point-of-care devices. Despite the significant number of tests performed, only few adverse events of swabbing have been reported [2-11]. Insertion of such applicators through natural orifices confers the possibility of foreign body retention with various consequences. This case report describes an unintended ingestion of an entire specimen collection applicator during self-swabbing.

## Case presentation

A 45-year-old male patient arrived at our otorhinolaryngology department for an emergency examination. Owing to the policy of his employing company, he was obliged to self-test for SARS-CoV-2 twice a week. Four hours before his arrival, the patient had attempted to perform a SARS-CoV-2 rapid antigen test (GenBody Inc, Cheonan, KR) at home by taking an OPS. Upon specimen collection, he felt a mild resistance and had a gag reflex, which resulted in the ingestion of the swab applicator.

The patient showed no signs of respiratory distress and had stable vital signs. He showed no apparent regurgitation of saliva. We performed a SARS-CoV-2 rapid antigen test (Hangzhou Clungene Biotech, CN) by taking an NPS and obtained a negative result.

During routine ear, nose, and throat examination, including flexible rhinopharyngolaryngoscopy, we found no foreign body in the upper respiratory tract or at the pharyngeal level.

**Figure 1:**
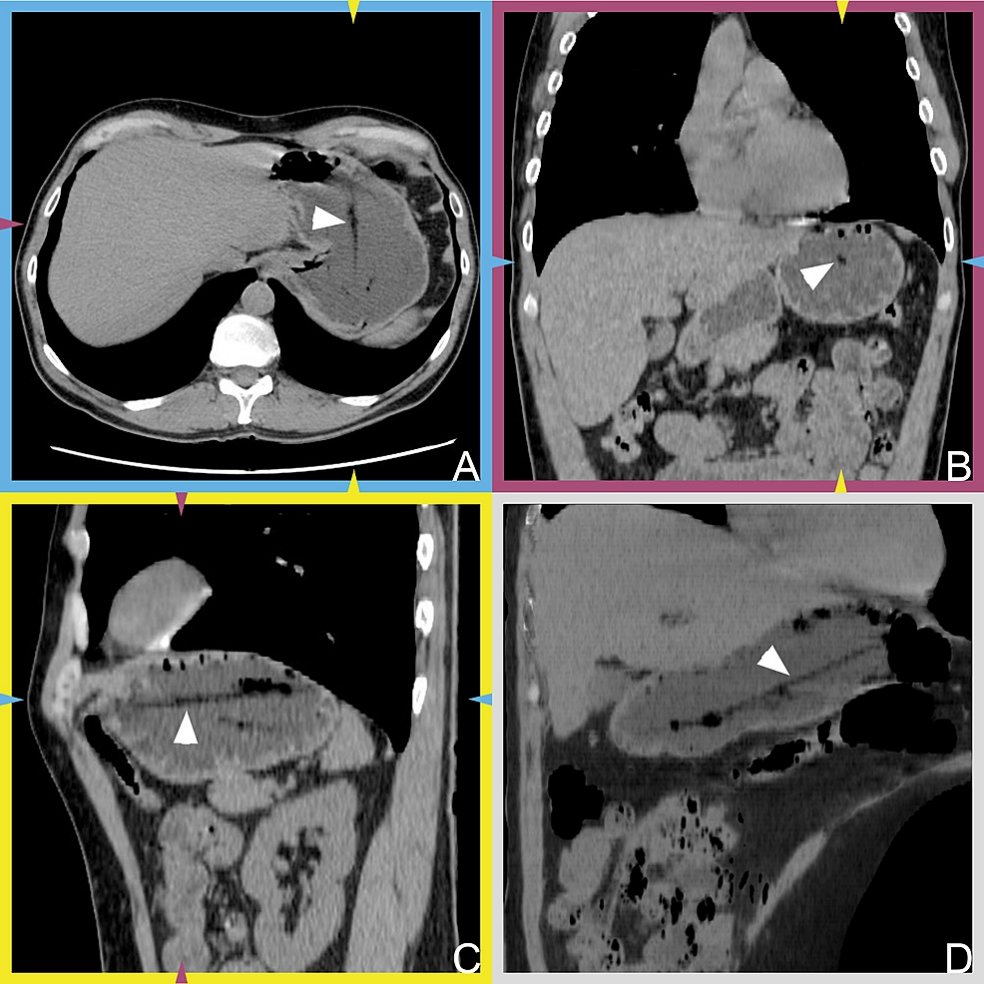
Multiplanar reconstructions of computed tomography scans. A–C: Multiplanar reconstructions of computed tomography images. A: axial plane, B: coronal plane, and C: sagittal plane. The elongated radiolucent area (white arrowheads) likely demonstrating the foreign body. The color-coded brackets and arrowheads indicate the corresponding planes. D: A minimum-intensity projection image generated from stacks, including sections of the foreign body. The oblique reconstruction was derived from sections acquired in a right recumbent position.

We performed thoracic and abdominal computed tomography (CT) scans in the supine and right lateral recumbent positions. Swab applicators are usually made of nylon. Thus, their radiopacity is not guaranteed without a documented radiopaque marking. In our case, we did not have the manufacturer’s description in hand. To overcome this problem, we asked the patient to drink water to fill the stomach with fluid. After proper windowing, a radiolucent band was visible in the stomach, suggestive of a foreign body (Figure 1). No other abnormalities were observable in the imaged regions.

**Figure 2:**
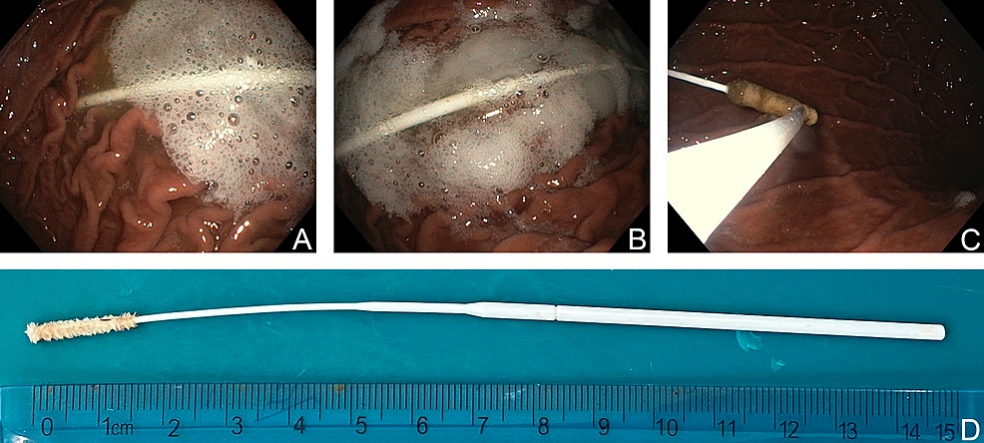
Gastroscopic removal of the foreign body. A–C: A series of endoscopic images visualizing the swab in the stomach. C: The tip of the foreign body is grasped with the loop. D: The applicator after removal. A 15-cm long object was removed intact.

In collaboration with the department of gastroenterology, we performed an urgent gastroscopy with a flexible endoscope (Olympus, JP). Despite four hours having elapsed since the incident, we still identified the swab stick intact in the stomach. After firmly catching the object with a polypectomy loop, it was withdrawn and removed safely (Figure 2). A second look showed no signs of mucosal damage or any residual foreign bodies along the esophagus and stomach. We examined the foreign body and confirmed that it had been removed without missing parts (Figure 2D). After eventless observation, the patient was discharged on the same day.

Informed consent was obtained from the patient for all the procedures and for the publication of information on his clinical course. All actions were made in accordance with the World Medical Association’s Declaration of Helsinki.

## Discussion

NPS and OPS are the most common routes of sampling for SARS-CoV-2 detection. Regardless of the location, correct collection technique is essential; otherwise low-quality specimens can lead to false-negative results [12,13]. Observations have defined NPS as superior to OPS, but an anatomical misconception exists regarding the nasal cavity even among healthcare professionals, resulting in poor-quality samples [12,14]. Wrong trajectory of the applicator can also be hazardous to the nasal structures. Complication rates are low, and they are minor and self-limiting in most cases, but severe adverse events have also occurred, including liquorrhea [4,5,7,9,10,15]. Asthma and respiratory allergy prevalence is still increasing [16]. These patients have a greater risk of involuntarily sneezing during the procedure resulting in the displacement or breakage of the applicator.

The nasal anatomy and breakpoint mechanism of the swab can result in a foreign body being retained [4-6,8,11]. Broken parts moving forward can either stick at the pharyngeal level or be aspirated or swallowed into the stomach, but only ingestions have been reported [3-5]. In one case, a nursing staff broke a wooden applicator during NPS, and the patient ingested the foreign body. Removal required immediate hospitalization and gastroscopic intervention [3].

Reports are scarce regarding the complications experienced after OPS [2,4,5]. We can assume that the spacious anatomy and broader trajectory made oropharyngeal swabbing safer than nasopharyngeal swabbing. The lower complication rate should be considered when designing self-testing kits for consumers, even though the sample quality gained through the transoral route is generally lower [14].

In the present work, we demonstrate an unusual case of foreign body ingestion in which the patient swallowed an applicator in one piece during a self-performed OPS. As the swab was made of nylon, we used indirect signs to localize the object on CT scans. We finally retrieved the foreign body with a flexible gastroscope and loop (Figure 2).

The tip of the current applicator is flexible (Figure 2). Too deep of an insertion might have resulted in downbending. The gag reflex increased the proximity of the esophageal inlet. Owing to the peristaltic movement, the stick was captured and moved into the stomach.

Rigid applicators, such as wooden ones, might be more appropriate for oropharyngeal testing, as a straight trajectory would be easier to maintain during the procedure. Thinner and flexible pieces might be more suitable for NPS. Confirmation of this theory will require controlled trials in the future.

One lesson learned from our case is that oropharyngeal testing can also be hazardous. Labeling of sticks with a radiopaque material would make the identification of swabs easier if involuntarily swallowed. Timing of the removal is crucial because further advancement of the foreign body may require surgical intervention [3]. Nevertheless, none of the technical developments can replace informative and educative user manuals designed especially for self-testing kits used by nonexpert subjects.

## Conclusions

For both NPS and OPS, errors can occur even in experienced hands. Oropharyngeal testing is an amenable approach for specimen collection but confers the risk of foreign body retention, as in the presented case. Radiopaque labeling of stick applicators would make their identification easier when involuntarily swallowed. To prevent subsequent consequences, foreign body removal should be performed promptly.

The increasing number of self-performed SARS-CoV-2 tests may increase the number of adverse events. Emergency departments should prepare for this tendency and provide multidisciplinary care.
